# Resistance Exercise Reverses Aging in Human Skeletal Muscle

**DOI:** 10.1371/journal.pone.0000465

**Published:** 2007-05-23

**Authors:** Simon Melov, Mark A. Tarnopolsky, Kenneth Beckman, Krysta Felkey, Alan Hubbard

**Affiliations:** 1 Buck Institute for Age Research, Novato, California, United States of America; 2 McMaster University, Department of Pediatrics and Medicine, Hamilton, Canada; 3 Center for Genetics, Children's Hospital Oakland Research Institute, Oakland, California, United States of America; Emory University, United States of America

## Abstract

Human aging is associated with skeletal muscle atrophy and functional impairment (sarcopenia). Multiple lines of evidence suggest that mitochondrial dysfunction is a major contributor to sarcopenia. We evaluated whether healthy aging was associated with a transcriptional profile reflecting mitochondrial impairment and whether resistance exercise could reverse this signature to that approximating a younger physiological age. Skeletal muscle biopsies from healthy older (N = 25) and younger (N = 26) adult men and women were compared using gene expression profiling, and a subset of these were related to measurements of muscle strength. 14 of the older adults had muscle samples taken before and after a six-month resistance exercise-training program. Before exercise training, older adults were 59% weaker than younger, but after six months of training in older adults, strength improved significantly (P<0.001) such that they were only 38% lower than young adults. As a consequence of age, we found 596 genes differentially expressed using a false discovery rate cut-off of 5%. Prior to the exercise training, the transcriptome profile showed a dramatic enrichment of genes associated with mitochondrial function with age. However, following exercise training the transcriptional signature of aging was markedly reversed back to that of younger levels for most genes that were affected by both age and exercise. We conclude that healthy older adults show evidence of mitochondrial impairment and muscle weakness, but that this can be partially reversed at the phenotypic level, and substantially reversed at the transcriptome level, following six months of resistance exercise training.

## Introduction

Human aging is associated with muscle atrophy (sarcopenia), weakness and functional impairment, which commence in the fourth decade of life with a rate of strength loss of about 1.0% per year, accelerating with each passing decade[1]. A functionally debilitating sarcopenia affects approximately 7% of adults over 70, and up to 20% over age 80 years[2], [3]. The estimated annual cost of sarcopenia-related health issues to the US health care system is more than 18 billion dollars annually[4]. The causes of organismal aging are complex, and a variety of common processes have been implicated in multiple tissues as being involved in driving the decline in function seen with increasing age. Some potential factors implicated in the functional decline of muscle include programmed cell death, oxidative stress, alterations in protein turnover, inflammation, hormonal dysregulation, disuse, and mitochondrial dysfunction[5]–[10]. Associations between mitochondrial dysfunction, the accumulation of mitochondrial DNA deletions, and sarcopenia have been seen in single fibers isolated from skeletal muscle in a number of species including humans [6], [11]–[14]. Recent support for a central role of mitochondria in degenerative changes associated with aging has come from a transgenic polymerase gamma “mutator” mouse that recapitulates many of the characteristic features of human aging[15], [16].

Several studies have evaluated transcript abundance in human skeletal muscle[8], [17], [18], some of which have implicated mitochondrial impairment in healthy adults[17], [18]. A recent report also indicated that mitochondrial dysfunction was a feature of aging[19]; however, the muscle tissue was harvested from patients undergoing surgery for illnesses that can alter mitochondrial mRNA abundance in skeletal muscle, such as cancer[20], [21]. Another study characterized the “molecular signature of sarcopenia” in healthy young and older adults, but did not report major alterations in the abundance of mitochondrial transcripts[8]. It has been suggested that transcriptome signatures of aging could be used to assess therapies meant to counter sarcopenia in humans, such as exercise[8], and this approach has been used to evaluate the beneficial effects of caloric restriction in murine skeletal muscle[22].

Long-term physical activity is associated with a reduction in morbidity and mortality in humans[23]. Resistance exercise can increase muscle strength, function and mass in older adults even into the 9^th^ decade of life[24]. An increase in muscle strength and hypertrophy are the main phenotypic outcomes of resistance exercise programs in younger adults[25]; however, resistance training in older adults can also increase mitochondrial capacity[26], and studies have shown that skeletal muscle atrophy and mitochondrial dysfunction often co-exist and may be causally related[6]. Furthermore, resistance exercise training can reduce markers of oxidative stress, and increase anti-oxidant enzyme activity in older adults[26], [27]. Life-long endurance exercise is associated with a “younger” transcriptome profile in cardiac muscle of inbred mice[28]. Endurance exercise training later in life has been shown to reverse some of the age-associated alterations in myosin heavy chain mRNA abundance[29]. Although the strength and functional benefits of resistance exercise training are well known[24], [30], [31], it is unclear whether training alters the age-associated alterations in the transcriptome profile of healthy older adults.

We report here that healthy older adults show a gene expression profile in skeletal muscle consistent with mitochondrial dysfunction and associated processes such as cell death, as compared with young individuals. Moreover, following a period of resistance exercise training in older adults, we found that age-associated transcriptome expression changes were reversed, implying a restoration of a youthful expression profile.

## Results

We first asked whether or not there was statistically significant differential gene expression in physiologically normal disease-free skeletal muscle between young and old individuals (Table S1). We identified 596 genes that were statistically significantly differentially expressed using a false-discovery rate (FDR) of 5% between the two age groups (Table S2).

**Figure 1:**
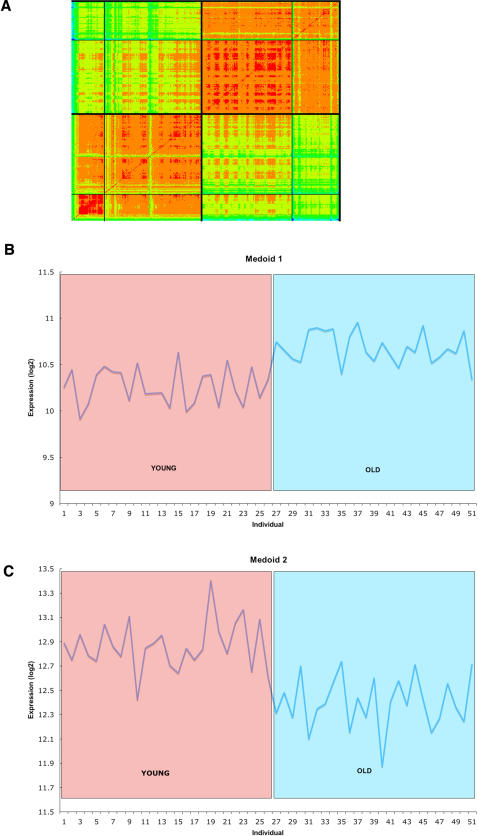
Differential Gene expression in physiologically normal individuals with age. Ordered distance matrix using the HOPACH algorithm[53] of significantly differentially expressed genes in young versus old skeletal muscle (FWER<0.05). Bold lines surround the first level of clustering, with the less bold lines indicating secondary clustering. Green to blue to purple is close to far. B) Medoid gene expression profile of a gene illustrating the expression behavior of cluster 1. C) Medoid gene expression of a gene illustrating the expression behavior of cluster 2.

We next examined co-ordinate regulation by clustering the list of 596 differentially expressed genes in healthy aged skeletal muscle. To display the clusters, we used the ordered distance matrix (where the genes are ordered by cluster and distance within cluster) returned by HOPACH and placed the cluster boundaries on this matrix. We can display the matrix not as numbers but as colors so genes that are close together are a “warm” color (red) and far apart a “cool” (green-blue). Though HOPACH provides hierarchical clusters, we just display the first level on this distance matrix. The procedure returned 2 main clusters of genes (in the first split of the data) associated with age (Figure 1), and each cluster could be further divided into two sub-clusters. Cluster 1 represents genes that increase in expression with age, while as Cluster 2 represents genes that decrease in expression with age. Figure 1b and 1c shows the medoid (the gene which most epitomizes the behavior of the cluster) for clusters one and two, showing a clear trend with age for genes associated with clusters one and two. To better understand the transcriptional response to age in normal skeletal muscle, we carried out GO analysis on the clusters and subclusters on the genes represented in Figure 1. No significant changes in GO terminology were common to all of the 290 genes in Cluster 1, or its subclusters, at 5% FDR, indicating there was no central theme at the gene ontology level to genes that *increased* in expression with age relative to young tissue. It is worth noting however, that statistically significant up-regulation of a number of genes involved in DNA repair, cell cycle control, transcription, and cell death were observed, consistent with previous reports of impairment of these processes with advancing age (Table S2, and [32]). In contrast, there was a common characteristic to the 306 genes whose transcriptional abundance decreased with age (Cluster 2): a striking association with energy metabolism and mitochondrial function (Table S3). We found up to a 39-fold enrichment in genes directly relating to mitochondrial metabolism and electron transport at a FDR of 5%, indicating that mitochondrial function declines markedly with age at the transcriptional level in skeletal muscle of physiologically normal individuals (Table S4). Examples of genes which decline with age in these ontologies are the gene encoding the beta subunit of succinyl-CoA synthase (SUCLA2[33]), the genes encoding the C subunit of succinate dehydrogenase (SDHC), and the ubiquinol-cytochrome C reductase hinge gene of complex III [34] (Figure S1). We next asked if genes which were significantly differentially expressed in healthy aging skeletal muscle, were also differentially expressed in aging muscle elsewhere in the body. We analyzed data collected from a variety of human muscles of diverse clinical backgrounds, as reported in Zahn et al[19], for differential expression with age at a 5% FDR. We detected no significant differential expression with age in this previously reported data, possibly due to the diverse clinical backgrounds of individuals used in that study, and a more stringent FDR[19]. Hence we were unable to compare age related gene expression in healthy skeletal muscle to the muscle reported by Zahn *et al*.[19].

**Figure 2:**
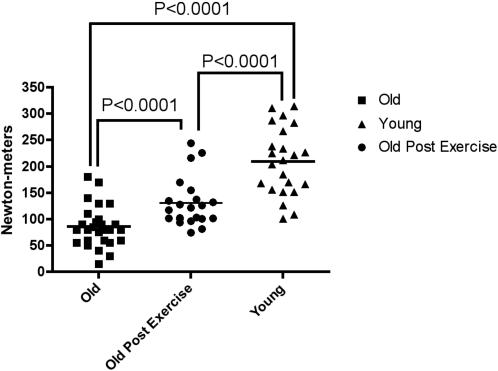
Exercise reverses a functional decline in the elderly. Each point represents an individual who underwent strength training as described in the methods. Young individuals had a greater capacity to lift the weights compared to older individuals (*p*<0.001, non parametric *t*-test). However, after 6 months training, the older individuals had a marked increase in their ability to carry out the exercise (*p*<0.0001, paired non-parametric *t*-test).

**Figure 3:**
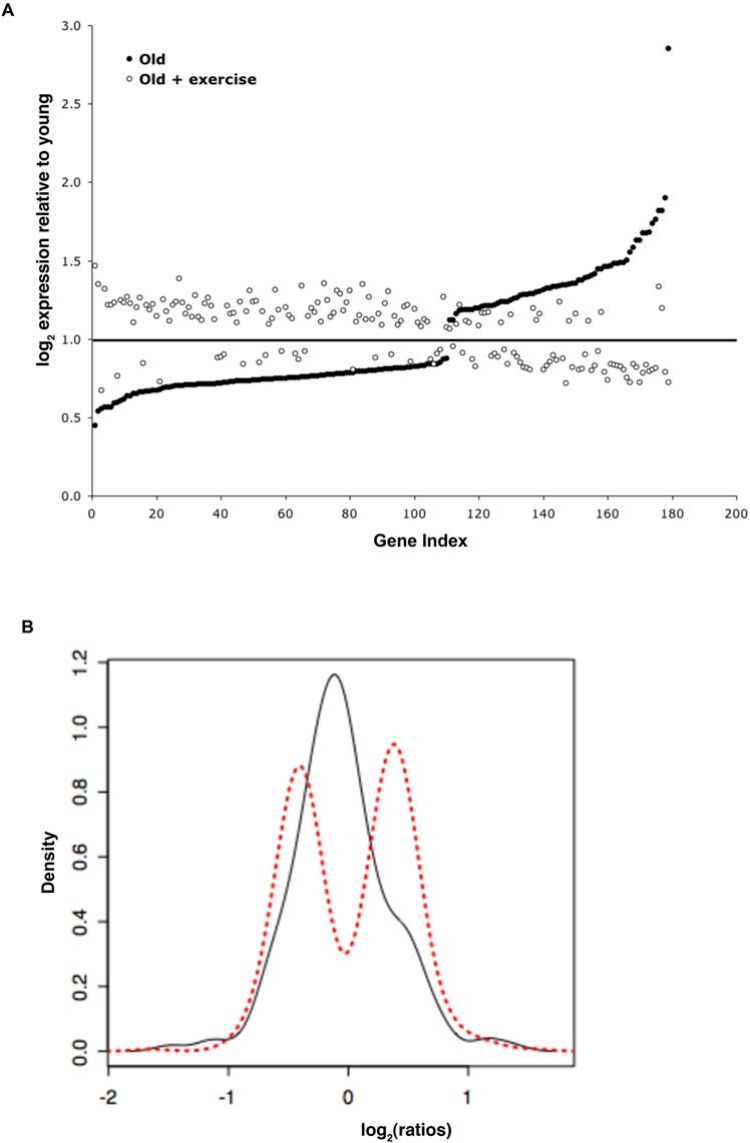
Gene expression changes associated with aging are reversed to youthful levels after 6 months of exercise training. Of the 596 genes associated with age in physiologically normal individuals, 179 of these were statistically significantly associated with a response to exercise at an FDR of 5%. a. Gene Index represents the 179 genes associated with age and exercise. The expression of these genes has been normalized relative to young values (young expression is represented by the dark line drawn across the graph at 1). Each genes average relative expression within the 14 older individuals can be seen relative to younger individuals. Genes that are downregulated with age show a marked reversal to youthful levels with exercise, and genes that are upregulated with age also show the same trend to return to youthful levels in association with exercise. b. Permutation testing of genes associated with exercise and age, to determine statistically significant reversals with exercise. The x axis (log_2_) represents the ratio of older subjects post-exercise gene expression compared to young individuals, with 0 being equivalent to “young” gene expression. This resulted in a *p*-value of <0.02. The conclusion is that the log_2_ ratios are much closer to 0 (expression is the same in old-exercise and young) than one would expect by chance (random draw of the 596 genes related to age).

We also measured peak muscle strength by determining maximal isometric knee extension torque in the right leg using a dynamometer (Biodex System 3, Biodex Medical Systems, Shirley, NY). We chose knee extension as the outcome variable because it correlates with functional capacity deficits[35], morbidity[36], and mortality[37] in sarcopenic older adults. We found that strength was 59% lower for older as compared to younger individuals (P<0.001, Figure 2), consistent with previous studies. However, upon regular exercise training, the older individuals were able to improve muscle strength by approximately 50%, to levels that were only 38% less than that of young individuals (Figure 2). In conjunction with this strength training, the older individuals had muscle biopsies taken from the same *vastus lateralis* muscle (separated by ∼3 cm) both before and after six months of strength training, facilitating the analysis of gene expression profiles of skeletal muscle associated with a functional improvement in strength in normal individuals. We determined which genes were differentially expressed with age, after exercise training, relative to young people at a FDR of 5%, and found a remarkable reversal of the expression profile of 179 genes associated with age and exercise training (Figure 3, Table S5). To demonstrate this, we used a plot of the distribution (smoothed kernel densities) of log_2_ ratio of expressions of old (post-exercise) over young for two sets among the aging genes: those that were significantly related to exercise and those that were not. We hypothesized that those related to exercise would result in expression more similar to young so the distribution of log_2_ ratios should be centered around 0 (equal expression) (Figure 3). However, genes unrelated to exercise should still have differential expression with regard to age (either down or up-regulated in older subjects so we should see two peaks, for these two distributions of relative expression centered around 0. These 179 genes were normalized in both directions, i.e. genes that were down-regulated with age were correspondingly up-regulated with exercise, while genes that were up-regulated with age, were down-regulated with exercise. Specifically, we randomly permuted the assignment of significance (with regards to exercise) among the 596 aging-related genes, and calculated the total distance (relative to young) of the resulting log_2_ ratios (old-exercise over young) of the genes significantly related to exercise by simply summing up squared log_2_ ratios (Figure 3). We then compared the permutation distribution of this sum to the observed sum. The *p* value is defined to be the proportion of times the sum of these squared log_2_ ratios was less than the observed sum of the log_2_ ratios of genes associated with exercise. Therefore a significant *p* value indicates that the older patients post exercise had expression levels much closer to younger subjects than one would expect by chance alone. We conclude that exercise improves muscle function in older people in association with transcriptional changes that recapitulate expression levels characteristic of young people (Figure 3).

**Figure 4:**
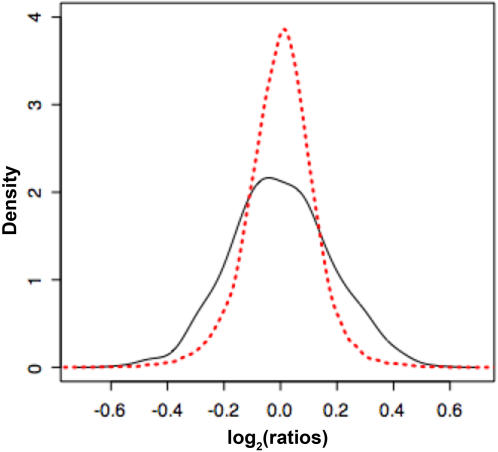
Exercise is more likely to affect “aging” genes than genes not associated with age. The x-axis (log_2_) represents the ratio of post over pre-exercise gene expression among older subjects. The dark solid line represents the distribution of log_2_ ratios of exercise (post/pre) among genes that are significantly associated with age. The red line represents the equivalent distribution for those genes not associated with aging. The *p*-value associated with the difference in distributions is less than 0.0001.

In order to verify that exercise is preferentially affecting the age-associated transcriptional profile, we examined differential expression (exercise – post vs pre) among both the 596 age-related genes and separately the 23758 remaining probes on the chip (Figure 4). The results indicate that exercise affects age-associated gene expression much more profoundly than non-age associated transcription (p<0.0001).

We next asked if the genes significantly associated with age and exercise were associated with specific strengths. We found no significant association of strength with gene expression up to an FDR cut-off of 25%, possibly due to the small variation between older individuals after training, limiting our power to associate specific genes with specific improvements in strength.

## Discussion

Our data strongly supports the concept that mitochondrial dysfunction is associated with aging in humans. The important and novel finding is that resistance exercise training reverses many aspects of the aging transcriptome signature. This implies that a functional improvement in aging muscle due to resistance exercise is associated with a global improvement in the molecular signature of aging particularly for transcripts related to mitochondrial function.

There are a number of lines of evidence supporting the hypothesis that mitochondrial dysfunction is a characteristic of human aging in skeletal muscle[6], [19], [38]. Studies have found lower mitochondrial enzyme activity[38], lower mitochondrial protein synthesis[39], an increase in mitochondrial DNA (mtDNA) deletions[26], a reduction in mtDNA content[38], and an increase in oxidative stress[27], in skeletal muscle from older adults. Importantly, a strong association has been found between skeletal muscle atrophy and the accumulation of mtDNA mutations and mitochondrial dysfunction in humans[6]. Although various aspects of the “mitochondrial theory of aging” have come under increasing scrutiny in the last several years[40], [41], two recent reports that transgenic animals with a mutation in polymerase γ (mutator mice) show many of the characteristics of human aging[9], [15], suggest that mitochondria may be involved in the pathogenesis of aging. Further support for a role of mitochondrial dysfunction in aging has come from transcriptome profiling studies in both animals and humans[17]–[19], [42]. Studies in humans using microarrays have also found lower skeletal muscle mRNA abundance for components of the mitochondria and energy yielding pathways in older versus younger adults[17], [18]. A recent study reported a coordinate down-regulation of many genes involved in mitochondrial structure and function in several tissues[19]. In contrast, a study reporting a “molecular signature of sarcopenia” in men did not report any up- or down-regulated mRNA species involved in either mitochondrial or energy yielding pathways[8]. Our data expands upon the latter studies in humans in a number of ways.

Firstly, we have compared a robust number of younger versus older adults who were very well characterized with respect to variables such as dietary and exercise background, the absence of medications (i.e., statins)[43] or diseases (i.e., cancer, type 2 diabetes)[44], that can alter mitochondrial function. Secondly, we have used a bioinformatics approach to discover new patterns of expression. Perhaps most importantly, we have demonstrated that resistance exercise training returned the transcriptome expression profile of the older individuals toward that of the younger adults.

Our observation of a “reversal” of the aging signature in skeletal muscle following six months of resistance exercise training is interesting from a number of perspectives. It is well known that resistance exercise training results in an increase in strength, function and muscle mass in younger and older adults[24], [30], [31], [45]. Furthermore, long-term habitual physical activity is associated with a lower risk of age-associated morbidity and mortality[23], [46]. We have previously shown that there is an increase in cytochrome c oxidase activity and a reduction in oxidative stress in older adults after several months of resistance exercise training[26], [27]. To our knowledge, the only other comparable data comes from mice that were inbred for their running capacity and the cardiac muscle transcriptome pattern for older “runners” was more similar to younger animals as compared with the less active older animals and the less active older animals were most different at the level of the mitochondria[28]. It is possible that our observation of a reversal of the mitochondrial based aging signatures could indicate that the response to exercise training was solely due to lower habitual exercise in the older adults. Arguing against this is the fact that the current study was specifically designed to avoid this confounder by selecting healthy, active, disease-free older adults and comparing them to similarly active younger adults (relatively inactive for age). In the future, it will be important to determine whether long-term or life-long exercise in humans can attenuate the transcriptome signature of aging using cross-sectional sampling in Masters athletes.

Finally, our data point to signature expression profiles that could potentially be used to screen for a variety of interventions that could reverse or return the aging signature towards that of younger adults. Candidate therapies or molecules that show promise could be entered into prospective trials to evaluate the efficacy in modulating the aging rate in skeletal muscle in otherwise physiologically normal adults.

## Materials and Methods

### Subject Characteristics and Exercise Testing

Muscle was acquired from a total of 25 healthy older adults and 26 younger adults. The muscle samples for the younger adults were obtained from recently completed studies in our laboratory with the pre-immobilization[10], and pre-exercise[47], samples used for comparison to the older adults. The younger adults were non-smokers, and were relatively inactive or participated in modest recreational activities and none were athletes. The older adults were relatively active (golfing, walking, gardening, tennis, cycling three or more times a week but were not competitive athletes) and healthy and volunteered to participate in a 26-week whole body resistance exercise-training program (see below). We specifically chose relatively well trained older adults and relatively sedentary younger adults (relative to others in their age group), such that we could study the effect of aging with subjects selected to be relatively well matched for activity across the age group and study the effects of aging *per se* and not merely inactivity. All older subjects underwent a thorough screening process before being admitted into the study to ensure absence of diseases that could alter mitochondrial function. Potential subjects were first screened by telephone, and then underwent a medical evaluation including review of past history and consent for participation from their family physician, a resting electrocardiogram (ECG), and a sub-maximal graded exercise test on a cycle ergometer with a post-exercise 12-lead ECG. Exclusion criteria included: evidence of coronary heart disease (by history and exercise test); congestive heart failure; hypertension; chronic obstructive pulmonary disease; diabetes mellitus; renal failure; orthopaedic disability precluding exercise training; and smoking. None of the subjects had previously participated in a structured resistance exercise program. The sex and age are given in Table S1.

Participants had their maximal isometric torque determined from the right leg using a dynamometer (Biodex System 3, Biodex Medical Systems, Shirley, NY). All testing was completed in the morning and the older adults were tested before training and between 48–72 h after the final training session (see below). A description of the testing methods has been provided previously by our group [10].

These studies were approved by the McMaster University and Hamilton Health Sciences Research Ethics Board and conferred to the principles of the declaration of Helsinki and all subjects gave written informed consent for participation.

### Exercise Training

Resistance exercise training was performed twice weekly on non-consecutive days (Monday+Thursday, or Tuesday+Friday) for 26 weeks in 14 of the older individuals listed in Table 1 (see Table S1 for details), under direct supervision by a research assistant. Prior to, and after each, training session, subjects were required to perform static stretching. Resistance exercise for each session consisted of 3 sets of 10 repetitions for each of; leg press, chest press, leg extension, leg flexion, shoulder press, lat pull-down seated row, calf raise, abdominal crunch, and back extension and 10 repetitions for arm flexion and arm extension. Training progressed from one set of each exercise at 50% of the initial 1 repetition maximum (1RM) to 3 sets at 80% of 1RM over the training period. Training logs were kept to record the volume and intensity of each session. The 1RM was re-evaluated every 2 weeks, and the training load was adjusted accordingly. All exercises were performed on plate-based strength training equipment (Universal Gym Equipment, Inc., Cedar Rapids, Iowa).

### Muscle Biopsy/Blood Sample

A muscle biopsy was taken from the *vastus lateralis* muscle of the right or left leg (randomized) before exercise or immobilization (young people, N = 26 total) and before (N = 25), and after (N = 14), the training period in older adults, ∼20 cm proximal to the knee joint using a 5mm Bergström biopsy needle. The muscle was dissected of fat and connective tissue, immediately frozen in liquid nitrogen, and stored at −80°C for subsequent analysis. All subjects were required to abstain from strenuous physical activity for 48 h prior to the muscle biopsy.

### RNA extraction, Microarrays, and Real-time PCR

Total RNA was extracted from human muscle with TRIzol reagent (Invitrogen, Carlsbad, CA) as described by our group[48]. Briefly, 25–50 mg of muscle was homogenized in 1 ml of TRIzol reagent at 4°C, left at room temperature for 5–10 min, followed by addition of 0.2 ml of chloroform, vortexing for 15 s, and centrifugation @ 12,000 rpm @ 4°C for 15 min. The supernatant was transferred to a fresh tube and mixed with 0.5 ml of isoproponal ethanol, stood at ∼22°C for 10 min, and centrifuged at 12,000 rpm at 4°C for 10 min. The RNA pellet was washed twice with 0.5 ml of 75% ethanol, air dried and dissolved in 14 µl of Depc-treated ddH_2_O with 2 µl aliquots stored at −80°C. The concentration and purity of the RNA was determined using a UV spectrophotometer (Shimadzu UV-1201; Mandel Scientific, Guelph, Ontario) at the absorbance of 260/280 nm. Measurements were done in duplicate and had an average coefficient of variation (CV) of <10%. The average purity (OD_260_/OD_280_) of the samples was >1.5 before DNAase treatment. RNA integrity was assessed in a randomly chosen subset of samples using agarose gel electrophoresis, and the OD ratio of 28S to 18S rRNA was consistently greater than 1 for each sample.

The resulting total RNA samples were further assessed for integrity prior to chipping using a Nanodrop Spectrophotometer and the Agilent Bioanalyzer Nano Chip System. Samples which passed this initial quality control assurance step were then amplified one round, using an RNA Amplification Kit (Ambion) according to the manufacturer's instructions. The resulting purified cDNA product of was resuspended in 18 µl and dried down using a speedvac. This was then converted to cRNA using an *in vitro* transcription kit according to the manufacturer's instructions (Roche), and again assessed for quality by using the Nanodrop and Bioanalyzer as described above. Labeled cRNA samples that passed this second round of quality control were then hybridized to Human Ref-8 BeadChips (Illumina) according to the manufacturer's instructions (approximately 23,000 genes), using equipment specified by the manufacturer (Illumina). Briefly, 850 ng biotin-labeled cRNA in 11.3 µl nuclease-free water was adjusted to 34 µl through the addition of 22.7 µl of 5∶3 HybE1 buffer/formamide. The sample was heated at 65°C for 5 min, allowed to cool to room temperature, and then immediately added to a single array of an 8-array Human Ref-8 BeadChip. Once all 8 samples were added to each BeadChip, it was sealed in a Hyb Cartridge and incubated for 16 h at 55°C with rotation in an Illumina hybridization oven (rotation setting 5). Following overnight hybridization, BeadChips were moved to a slide rack and serially washed using gentle rotation in glass staining dishes filled with a) 250 ml Illumina Wash Buffer×5 min, b) 250 ml 100% ethanol×10 min, c) 250 ml Illumina Wash Buffer×2 min. BeadChips were then blocked for 10 min in 4 ml Block E1 buffer (Illumina), followed by staining for 10 min in 1 µg/ml Streptavidin-Cy3 conjugate (GE Healthcare) in Block E1 buffer. Stained BeadChips were finally washed using gentle rotation in a glass staining dish filled with 250 ml Illumina Wash Buffer×5 min. BeadChips were dried by centrifugation at 280×***g*** for 4 min and stored in a light-tight box until reading.

Recent studies by the MAQC (microarray quality control) project, show substantial agreement between illumina microarray data and real-time PCR data on the same samples[49]–[51]. However, although there exists ample precedent for the veracity of microarray data without “independent” confirmation via real-time PCR (which can suffer from a number of significant technical issues [52]), we carried out real-time PCR validation of a number of genes to confirm key aspects of our study (Figure S2). The RT-PCR was completed for 2 of the genes on the microarray (Figure S2) using a TaqMan® real-time method. Briefly, samples were treated with DNase I for 25 min to remove any contaminating DNA. Primers and probe to each target gene were designed based on the cDNA sequence in GenBank (http://www.ncbi.nlm.nih.gov/entrez/query.fcgi) with primer 3 designer (http://frodo.wi.mit.edu/cgi-bin/primer3/primer3_www.cgi). All target gene probes were labeled with FAM at their 5′ ends and BHQ-1 at their 3′ ends. Duplex RT-PCR was performed on an iCycler real-time PCR system (Bio-Rad Laboratories, Hercules, CA) in the One-step TaqMan® RT-PCR Master Mix Reagents (Roche, Branchburg, New Jersey) according to the manufacture's instruction with target gene primers and probe and internal standard gene primers and probe in the same reaction.

### Array Reading

Processed arrays were read using a BeadStation array reader (Illumina) according to the manufacturer's instructions. Scan settings were for single color (green) scanning: Factor = 2.506, PMT = 561, Filter = 100%.

### Bioinformatics and Statistics

After quantile normalization (using *limma* package in R) on the age arrays (25 young and 26 older subjects, Table S1), we first determined the number of genes significantly differentially expressed with age, by performing on a probe-by-probe basis, 24354 two-sample *t*-tests. Illumina chips routinely have approximately 30 independent replicates of each gene (technical replicates) on the chip, and we used the average of these replicates. It is worth emphasizing that this provides a high degree of confidence for the estimation of each genes abundance, as 30-fold technical replication is many times that of other methodologies for measuring gene abundance such as real-time PCR. The gene expression data reported here is deposited in GEO (http://www.ncbi.nlm.nih.gov/geo/).

We chose the significant genes to control the familywise error rate (FWER) at 5% using Holm's step-down method – we used FWER because we desired to have a low probability of any false positives among this list. Among these genes, we examine the association of exercise using the paired arrays. We had 14 older subjects with both pre and post exercise regime samples (and corresponding microarrays), and were therefore able to 1) derive the log_2_ expression values on the 28 arrays using quantile normalization and 2) created a new data set which was the difference of the log_2_ expressions for each subjects or log_2_(post/pre), resulting in now 14 vectors (each of length the number of significant age related genes) of vectors, one for each pair (post vs. pre) of observations on the same subject. To determine which of the age-related genes were related to exercise, we used paired *t*-tests and adjusted for multiple testing using the FDR and the Benjamini and Hochberg method. Among those genes with an adjusted q-value (based on FDR) of <0.05, we used hierarchical clustering (based on the HOPACH algorithm to find groups of genes with similar profiles across the subjects. An advantage of HOPACH clustering is that it provides a criterion for choosing the optimal number of clusters for each level of the hierarchy, using the so-called “silhouette” statistic [53]. In addition, we examined this subset of genes to find the set that made the older subjects in the post exercise group most like the younger subjects in the original set of arrays. Specifically, we created a new data set of both young and older subjects post-exercise. After normalization and reducing the probes to the number of genes “significantly related to exercise and age” we looked at the ratio of post exercise older subjects vs. young subjects, only among those genes that were related to age (old versus young, 596 genes). We examined distribution of the ratios (among genes) in two groups of age-related genes: those that were differentially expressed after exercise and those that were not significantly related to exercise. We then plotted the smoothed densities (smoothed histograms) of the log_2_ ratios of these two groups. To test the significance of this plot, we used a permutation test (permuting the designation of an “exercise related gene”) and as summary test statistic the average squared log_2_ ratios (the average Euclidean distance from the log_2_(1)). To analyze the data from Zahn, et al., we attempted to mimic the analyses reported in their paper. We performed adjusted linear regressions of log_2_(expression) versus age from their data which was obtained from a public database; we performed an analysis adjusting only for age and one adjusting for both age and tissue type. Again, after ordering the genes by statistical significance (using the standard Wald test on the slope), we use Benjamini and Hochberg to derive so-called *q*-values (or the estimated false discovery rate using the corresponding raw *p*-value as the cut-off). Our results for both showed no genes differentially expressed (in any analysis) using an FDR cut-off of 5%.

To test whether or not the genes that were significantly differentially expressed with age were being restored to youthful levels via exercise, we performed a permutation test to determine if this apparent enrichment (in genes related to exercise) was significant. Specifically, we randomly permuted the assignment of significance (with regards to age – so a vector of 596 one's and 23758 zero's) among all 24354 probes and calculated the distance of the resulting log_2_ ratios (post/pre exercise) of the genes by simply summing up squared log_2_ ratios of those genes randomly assigned to significant age genes. We then compared the permutation distribution of this sum to the observed sum, the *p* value is defined as the proportion of times the sum of these squared log_2_ ratios (post/pre) was greater than the observed sum of the log_2_ ratios (post/pre) of genes associated with age. A small *p*-value would indicate that many more of the genes associated with age are also associated with exercise than one would expect by chance.

### Gene ontology analysis

In the lists of genes that were significantly differentially expressed with age or exercise in our study, we carried out GO-analysis to determine the relative enrichment of genes with common or related functionalities to gain insight into biological processes mediated by age or exercise. This was carried out using the web interface driven GoMiner tool using a False discovery rate of 5% [54]. (http://discover.nci.nih.gov/gominer/index.jsp).

## Supporting Information

Table S1 Age and Sex of individuals used in these studies. The subset of individuals who were subjected to exercise training is also shown “Post exercise”.
(0.03 MB XLS)

Table S2 Differential Gene expression as a function of age in physiologically normal individuals. Differentially expressed genes are ranked from most significantly changed to least significant, with a cutoff at 5% FDR.
(0.29 MB XLS)

Table S3 GO miner analysis of the genes listed in Supplemental Table 2. For a complete description of the column headers, see http://discover.nci.nih.gov/gominer/hi-thruput-defs.jsp.
(0.51 MB XLS)

Table S4 Go miner analysis of genes with decreased abundance and age (Cluster 2). Column headers are as described in http://discover.nci.nih.gov/gominer/hi-thruput-defs.jsp.
(0.04 MB XLS)

Table S5 Exercise reverses gene expression in older adults to that of younger adults. 179 genes are shown relative to the average of young expression both before and after 6 months of exercise training. The readings are the average of the 14 adults listed in Supplemental Table 1, Post exercise. NB, Figure 3a is a diagram of this data.
(0.12 MB XLS)

Figure S1 Mitochondrial genes decline with age. Shown here are three genes which encode mitochondrial proteins, all of which decline with age. A) the beta subunit of succinyl CoA synthase (SUCLA2); B) the C subunit of succinate dehydrogenase (SDHC); C) the ubiquinol-cytochrome C reductase hinge gene of complex III (UQCRH).
(0.41 MB DOC)

Figure S2 Real time validation of 2 genes from the list of genes identified via microarray expression profiling.
(0.05 MB DOC)
