# Supplementary figures and images for: Resistance Exercise Reverses Aging in Human Skeletal Muscle
Source: PLoS One. 2007 May 23;2(5):e465. doi: 10.1371/journal.pone.0000465 (PMC1866181; doi:10.1371/journal.pone.0000465)

**A)**


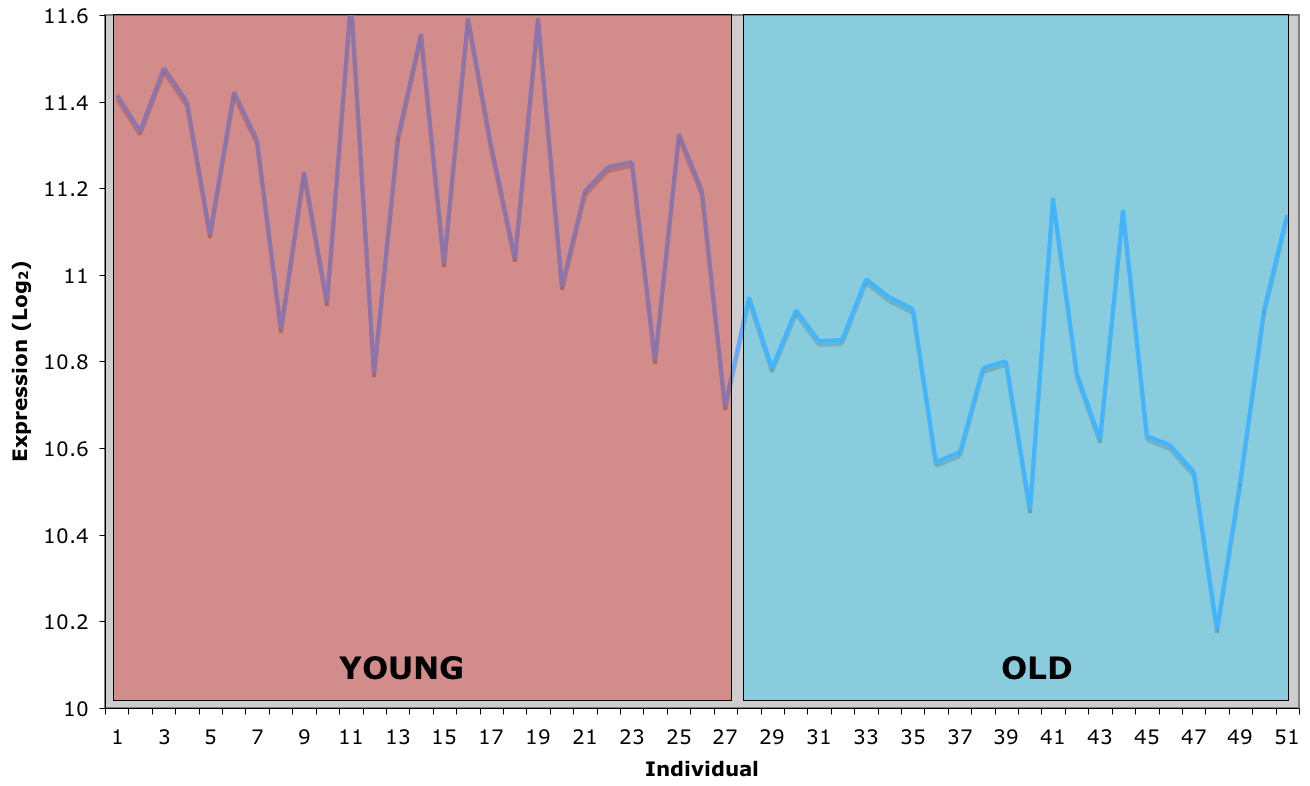


**B)**


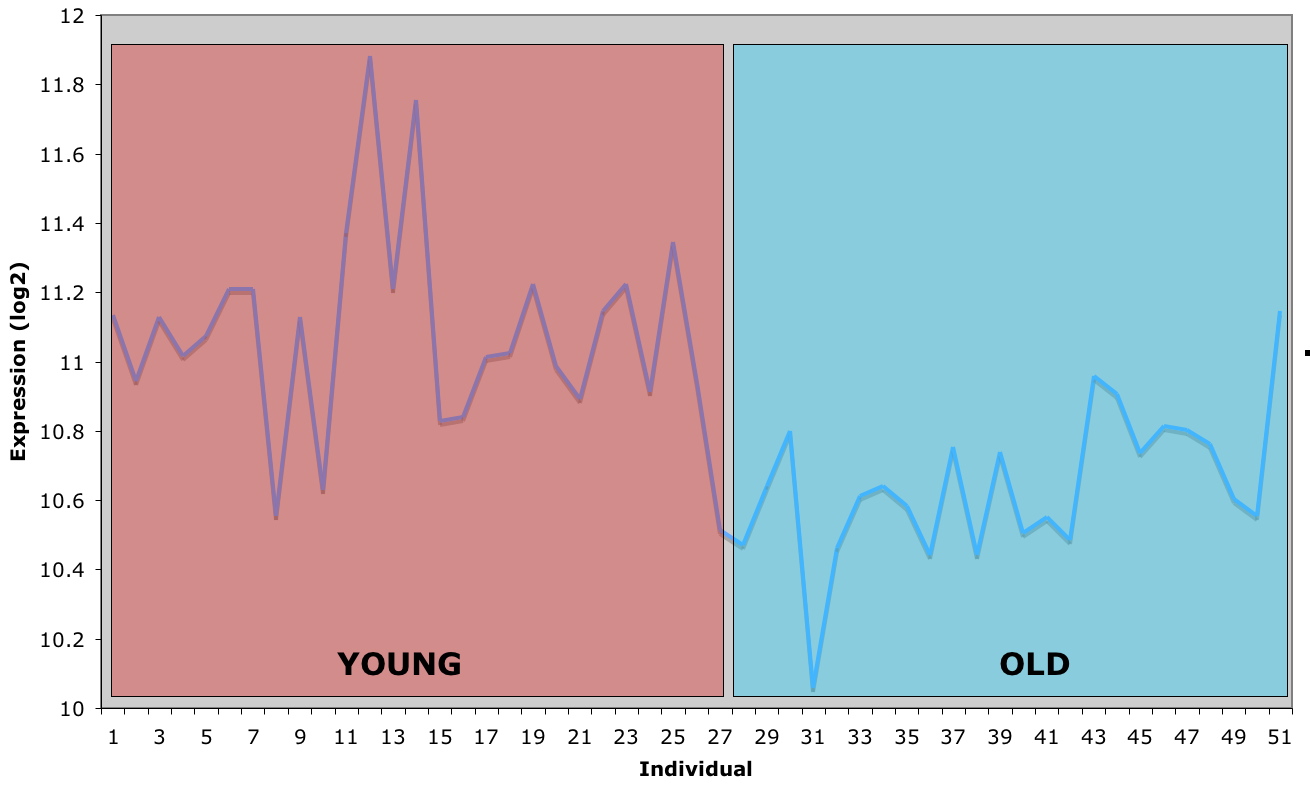


**C)**


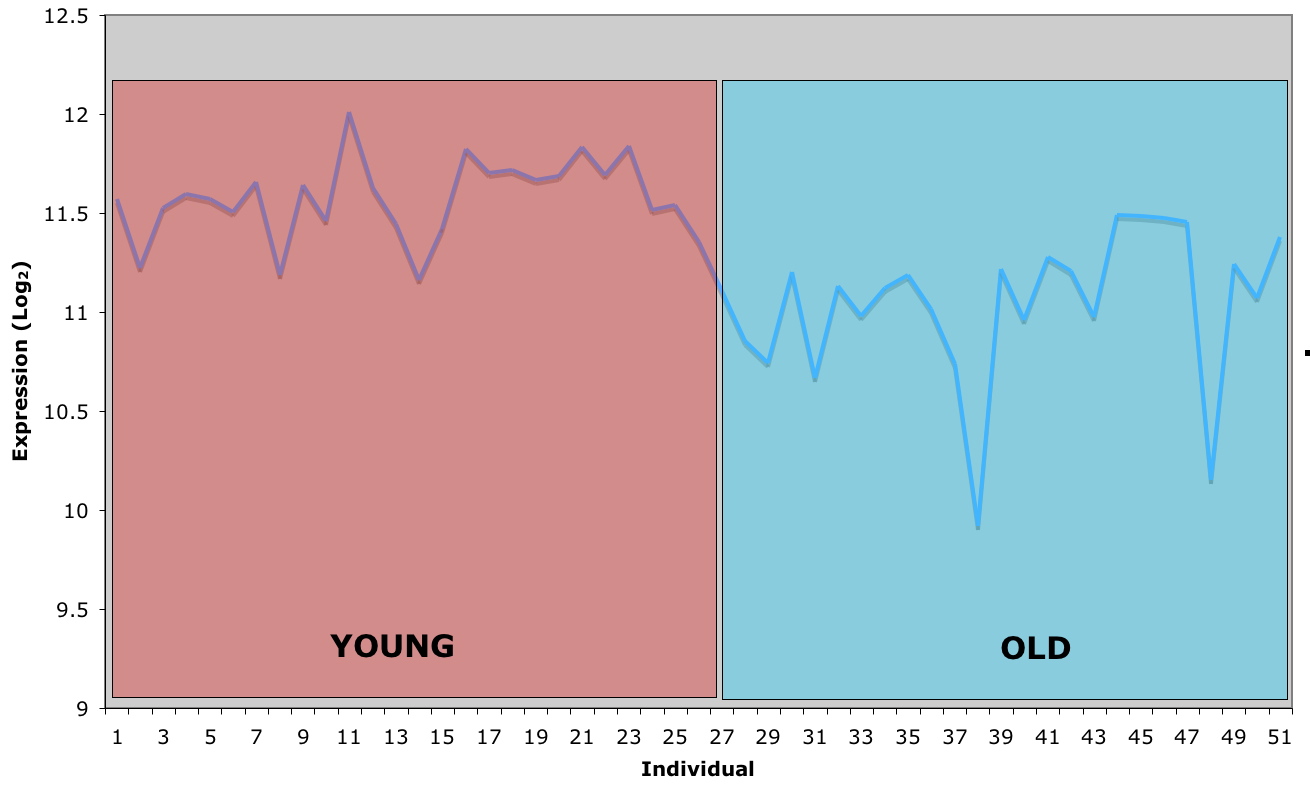

Supplement: Figure S1 — Mitochondrial genes decline with age. Shown here are three genes which encode mitochondrial proteins, all of which decline with age. A) the beta subunit of succinyl CoA synthase (SUCLA2); B) the C subunit of succinate dehydrogenase (SDHC); C) the ubiquinol-cytochrome C reductase hinge gene of complex III (UQCRH). (0.41 MB DOC) [file pone.0000465.s006.doc]
