# Supplementary material for: Resistance Exercise Reverses Aging in Human Skeletal Muscle
Source: PLoS One. 2007 May 23;2(5):e465. doi: 10.1371/journal.pone.0000465 (PMC1866181; doi:10.1371/journal.pone.0000465)
Supplement: Figure S2 — Real time validation of 2 genes from the list of genes identified via microarray expression profiling. (0.05 MB DOC) [file pone.0000465.s007.doc]

Confirmation of microarray data

For each of the genes for which RT-PCR data was collected, we examined whether we found a significant relationship with age and whether the expression correlated with the microarray data generated by our Illumina studies. Specifically, from the 51 subjects, we choose 39 for RT-PCR analyses (17 Older and 22 Younger subjects, based on tissue availability).

RT-PCR boxplots of expression by age for A (FABP3) and B (ADH1B)

**A**

**B**

First, we simply examined whether the expression direction (and significance) observed in the original analysis corresponded to that for the RT-PCR. We did this analysis for 2 genes on the list of significantly differentially expressed with age: FABP3 and ADH1B. To test with the same pattern of relative expression occurred, we simply carried out boxplots by age (see above) and performed two-sample t-tests to test for the significance of the apparent differential expression.

For FABP3, originally older subjects were significantly downregulated (ratio of expression approximately 0.63 Old/Young, adjusted q-value based on FDR < 0.001). For the RT-PCR results, we also see lower expression in the young (ratio of 0.51 Old/Young) which is also significantly differentially expressed (p < 0.001). For ADH1B, the Illumina data showed up-regulation in the older subjects (ratio = 1.90, adjusted FDR q-value < 0.001) and the rt-PCR results confirmed this pattern of differential expression (ratio of 3.1 Old/Young, p = 0.02). Therefore, our initial results obtained by microarray are confirmed.

In addition, we simply looked at the correlation of expression between our microarray results (Illumina) versus our RT-PCR results for these two genes (specifically, log_2_ expression). We see reasonable correlation for both FABP3 (0.67) and ADH1B (0.59), lending further confidence to our overall methodology.

RT-PCR correlation (Illumina microarray vs RT-PCR) plots of log_2_(expression) for A (FABP3) and B (ADH1B)

**A**

**B**
